# Prevalence and Correlates of Pain Among Nurses in Poland: A Cross-Sectional Study

**DOI:** 10.3390/healthcare14193139

**Published:** 2026-09-22

**Authors:** Agata Ważna, Małgorzata Dziechciaż, Amir Khorram-Manesh, Krzysztof Goniewicz, Ahmed M. Al-Wathinani, Mariusz Goniewicz

**Affiliations:** 1 Faculty of Health Sciences, The State University of Applied Sciences in Jaroslaw, 37-500 Jaroslaw, Poland; 2 Department of Surgery, Institute of Clinical Sciences, Sahlgrenska Academy, Gothenburg University, 405 30 Gothenburg, Sweden; 3 Center for Disaster Medicine, University of Gothenburg, 405 30 Gothenburg, Sweden; 4 Gothenburg Emergency Medicine Research Group (GEMREG), Sahlgrenska Academy, Gothenburg University, 405 30 Gothenburg, Sweden; 5 Department of Security, Polish Air Force University, 08-521 Deblin, Poland; k.goniewicz@law.mil.pl; 6 Department of Emergency Medical Services, Prince Sultan Bin Abdulaziz College for Emergency Medical Services, King Saud University, Riyadh 11451, Saudi Arabia; 7 Department of Emergency Medicine, Medical University of Lublin, 20-081 Lublin, Poland; mariusz.goniewicz@umlub.pl

**Keywords:** nurses, pain, health behaviors, occupational health, professional experience, analgesic use, activity limitation

## Abstract

**Background:** Pain is a prevalent occupational health concern among nurses and may affect functional capacity and work performance. However, evidence from Central and Eastern Europe remains limited, particularly regarding behavioral and occupational correlates of pain. **Objectives:** To characterize pain among practicing nurses and examine associations between health-related behaviors, occupational characteristics, and distinct pain-related outcomes. **Methods:** A cross-sectional, questionnaire-based study was conducted among 580 nurses in southeastern Poland. Pain was assessed using the four-item Laitinen Pain Scale (LPS), and health-related behaviors were evaluated using the Health Behavior Inventory (HBI). Descriptive statistics, univariable analyses, and multivariable logistic regression were used to examine associations with pain intensity, pain frequency, analgesic use, and activity limitation. **Results:** More than 80% of participants reported pain. Among the HBI domains, the lowest scores were observed for Health Practices (mean 19.1 ± 4.4). Compared with nurses with <10 years of professional experience, those with 10–29 years had higher odds of reporting pain intensity (OR = 2.94; 95% CI: 1.29–6.64), pain frequency (OR = 3.74; 95% CI: 1.54–9.08), and analgesic use (OR = 2.38; 95% CI: 1.09–5.16). Lower Health Practices scores were associated with higher odds of pain intensity (OR = 2.00; 95% CI: 1.17–3.41), pain frequency (OR = 2.20; 95% CI: 1.21–3.99), and analgesic use (OR = 1.78; 95% CI: 1.09–2.91). Lower Positive Mental Attitude scores were associated with activity limitation (OR = 1.78; 95% CI: 1.10–2.87). **Conclusions:** Pain was frequently reported among nurses in this regional sample and was associated with professional experience and selected health-behavior domains. The cross-sectional design precludes causal inference. Longitudinal and interventional studies are needed to clarify these relationships and determine whether occupational or behavioral interventions can reduce pain or improve functional outcomes.

## 1. Introduction

Pain is among the most prevalent health complaints affecting people worldwide. Global surveys involving 1.6 million respondents from 146 countries have demonstrated a rising trend in pain prevalence, from 26.3% in 2009 to 32.1% in 2021 [1]. Chronic pain is increasingly recognized not merely as a symptom of disease, but as a distinct pathological condition, one that constitutes a source of suffering and profoundly affects patients’ quality of life [2].

Nurses are particularly vulnerable to pain, especially work-related musculoskeletal pain. International meta-analyses indicate a high prevalence of musculoskeletal complaints in this occupational group [3,4]. These symptoms are clinically and occupationally relevant because they may contribute to functional limitations, sickness absence, and impaired work performance [5,6,7].

Key occupational factors associated with pain among nurses include manual patient handling and prolonged or awkward postures [8]. In addition to occupational exposures, health-related behaviors may be relevant to nurses’ overall health and well-being. Previous studies have identified challenges in maintaining regular physical activity, healthy dietary practices, adequate rest, and other health-promoting behaviors in nursing populations [9,10,11,12,13]. These behaviors may therefore represent an additional dimension to consider alongside occupational characteristics when examining pain among nurses.

From a biopsychosocial perspective, pain among nurses may be understood as reflecting the interplay of occupational exposures, health-related behaviors, and psychological factors [14]. Health practices such as physical activity, adequate rest, and sleep may be relevant to physical recovery and functional capacity, whereas psychological factors, including stress and coping, may be associated with pain perception and pain-related activity limitation [15]. Dietary and preventive behaviors may additionally reflect broader patterns of health maintenance. Importantly, some of these relationships may be bidirectional, as pain itself may interfere with sleep, physical activity, and other health-related behaviors [16]. Thus, rather than assuming a causal effect of health behaviors on pain, the present study considers individual health-behavior domains as potentially relevant correlates of different dimensions of pain.

Although pain among nurses has been extensively studied, previous research has predominantly focused on its prevalence and on individual occupational, physical, or psychosocial correlates. Recent studies have also examined selected behavioral and psychological factors associated with pain among nurses [17,18,19]. However, evidence remains limited on whether broader patterns of health-related behaviors are differently associated with distinct dimensions of pain. In particular, the relationships between the individual domains of the Health Behavior Inventory (HBI), proper eating habits, preventive behaviors, positive mental attitude, health practices, and specific pain outcomes, including pain intensity, pain frequency, analgesic use, and activity limitation, have not been sufficiently characterized. Examining these domains and pain outcomes separately may provide a more differentiated understanding of the behavioral correlates of pain than analyses based on overall health behavior or pain measures alone.

Accordingly, the present study aimed to characterize pain among practicing nurses and to examine the associations between individual HBI domains and distinct dimensions of pain assessed using the Laitinen Pain Scale (LPS), while also considering relevant sociodemographic and occupational characteristics. By examining individual behavioral domains alongside separate pain outcomes, the study sought to provide a more differentiated assessment of the correlates of pain in this occupational group.

## 2. Materials and Methods

### 2.1. Study Design

This study employed a cross-sectional, questionnaire-based design aimed at assessing the prevalence and correlates of pain among practicing nurses, as well as its association with health-related behaviors. Participation was entirely voluntary and non-incentivized, and no interventional or experimental procedures were involved.

### 2.2. Setting and Participants

The research was carried out in the Podkarpackie Voivodeship, southeastern Poland, between July 2023 and March 2025. Data collection was completed before the statistical analysis and manuscript preparation. The extended recruitment period reflected the inclusion of multiple healthcare institutions and the phased organization of survey distribution.

According to national statistics from 2022, approximately 12,800 nurses were employed in direct patient care within this region [20]. Based on this population size, a minimum sample size of 373 was estimated assuming a 95% confidence level and a 5% margin of error. The final study sample comprised 580 nurses. This calculation was used to assess sample-size adequacy and should not be interpreted as establishing the representativeness of the non-probability sample. Respondents represented a variety of healthcare institutions, including hospitals, primary care clinics, sanatoria, nurseries, and long-term care facilities (nursing homes, residential care centers, and home-based nursing services).

The study employed a non-probability convenience sampling approach. Paper and electronic questionnaires were distributed within participating healthcare institutions by administrative staff or ward managers, and participation was fully voluntary and anonymous. The participating institutions did not systematically record the number of questionnaires distributed or the number of potentially eligible nurses who received an invitation to participate. Consequently, the denominator required to calculate a response rate was unavailable, and the response rate could not be determined. A total of 580 completed and eligible questionnaires were included in the analysis.

#### Inclusion and Exclusion Criteria

Eligible participants were registered nurses actively employed in healthcare institutions, including hospitals, outpatient clinics, and long-term care facilities, and aged 21 years or older. Participation was voluntary and anonymous. Nurses who were not currently practicing (e.g., retired, on leave, or students) or who returned incomplete questionnaires (more than 10% missing data) were excluded from the analysis.

### 2.3. Research Instruments

Two standardized instruments were used: the Laitinen Pain Scale for pain assessment [21] and Juczyński’s Health Behavior Inventory for evaluation of health-related behaviors [22]. Additional data were collected on sociodemographic and professional characteristics (age, sex, education, place and duration of employment, and secondary job holding).

To assess the reliability of the measurement tools, we examined internal consistency coefficients for both instruments. The LPS has been used in previous studies conducted in Polish clinical and research populations. In the present sample of practicing nurses, the LPS demonstrated satisfactory internal consistency (Cronbach’s α = 0.81). This reliability analysis was based on data from the present study sample and did not constitute a formal validation of a Polish version of the LPS. Juczyński’s HBI has shown good reliability in the original Polish standardization, with a Cronbach’s alpha of 0.85 for the total score and subscale values ranging from 0.60 to 0.65. In our study, internal consistency was α = 0.88 for the total HBI score, with Cronbach’s alpha values for the four subscales ranging from 0.62 to 0.70, indicating acceptable reliability.

### 2.4. Laitinen Pain Scale

The LPS is a self-report instrument assessing four dimensions of pain, each rated from 0 to 4. Pain intensity is scored as 0 = no pain, 1 = very mild, 2 = moderate, 3 = severe, and 4 = unbearable pain. Frequency of pain episodes is scored as 0 = never, 1 = rarely, 2 = occasionally, 3 = often, and 4 = constantly. Frequency of analgesic use is scored as 0 = never, 1 = rarely, 2 = occasionally, 3 = often, and 4 = always. Limitation of daily activities is scored as 0 = none, 1 = partial limitation, 2 = limitation interfering with work, 3 = limitation preventing work, and 4 = limitation preventing normal functioning.

### 2.5. Health Behavior Inventory

The HBI comprises four domains: Proper Eating Habits, Preventive Behaviors, Health Practices, and Positive Mental Attitude [22]. The instrument consists of 24 statements, with six items assigned to each domain, rated on a five-point Likert scale. The total score ranges from 24 to 120, with higher scores indicating a higher level of health-promoting behavior.

### 2.6. Statistical Analysis

Descriptive statistics were presented as means ± standard deviation (SD), medians, and interquartile ranges (Q1–Q3) due to the non-normal distribution of several variables (Shapiro–Wilk test). Categorical variables were summarized as counts and percentages.

Associations between continuous variables (e.g., age and years of professional experience) were assessed using Spearman’s rank correlation coefficient (R). Group comparisons were performed using the Chi-square test with Yates’ correction for small expected counts. Each of the four LPS dimensions was analyzed as a separate binary outcome: pain intensity (0 = no pain vs. ≥1 = any pain), pain frequency (0 = never vs. ≥1 = any reported pain frequency), analgesic use (0 = never vs. ≥1 = any analgesic use), and activity limitation (0 = none vs. ≥1 = any activity limitation). The original 0–4 distributions of all four LPS dimensions are presented descriptively in the Results.

The following variables were analyzed: age group (21–39, 40–59, ≥60 years), sex, education (medical college, bachelor’s, master’s degree), workplace (hospital, outpatient clinic, long-term care facility; other workplaces were excluded due to very small sample sizes), years of experience (<10, 10–29, ≥30 years), additional employment (yes/no), and HBI domain scores dichotomized at the sample median (relatively lower vs. higher scores) [23]. All predictors entered into the logistic regression models were analyzed as categorical variables; therefore, assessment of linearity in the logit for continuous predictors was not applicable. Candidate variables for the multivariable logistic regression models were screened using univariable analyses. Variables with *p* < 0.10 in the univariable analysis were considered for inclusion in the corresponding multivariable model. This threshold was used as an exploratory screening criterion rather than as evidence of statistical significance. Multivariable logistic regression was subsequently used to estimate adjusted associations between the included variables and each pain outcome [24]. Age and years of professional experience were strongly correlated (Spearman’s R = 0.895, *p* < 0.001). To avoid including two closely related measures of occupational seniority in the same multivariable models, professional experience was retained as the occupationally more directly interpretable variable, whereas age was excluded. Multicollinearity among the variables retained in the multivariable models was subsequently assessed using variance inflation factors (VIFs).

For the regression analyses, HBI domain scores were dichotomized at the sample median to provide an interpretable comparison between participants with relatively lower and higher levels of health-promoting behaviors within the study sample. The median-based categories were used as an analytical operationalization and should not be interpreted as validated clinical cut-offs. We acknowledge that dichotomization reduces the information contained in the original continuous scores and may reduce statistical power.

Missing HBI responses were minimal and were handled according to the instrument manual: if no more than one item was missing within a subscale, the missing value was replaced with the mean of the remaining items. Questionnaires with more than 10% missing responses were excluded from the analysis. All variables retained in the multivariable models had VIF values < 2.5, indicating no problematic multicollinearity among the included predictors.

The overall sample comprised 580 participants. No separate a priori sample-size calculation was performed for each logistic regression model; the original sample-size calculation was based on the regional nursing population. No additional formal goodness-of-fit diagnostics were reported for the logistic regression models.

A *p*-value of <0.05 was considered statistically significant. All analyses were two-tailed and performed using IBM SPSS Statistics version 28.0 (IBM Corp., Armonk, NY, USA).

### 2.7. Ethical Considerations

The study complied with national and institutional ethical standards as well as the principles of the Declaration of Helsinki. According to Polish law, non-interventional survey studies that do not include medical procedures are exempt from formal ethical approval. This was confirmed by the Bioethics Committee of the Poznan University of Medical Sciences (Statement No. KB-827/23). Participation was voluntary and anonymous, and all respondents provided informed consent prior to inclusion.

## 3. Results

A total of 580 nurses from southeastern Poland participated in the study. The following subsections present the demographic and occupational characteristics of the study group, the distribution of health-related behaviors, the results of pain assessment based on the Laitinen Pain Scale, and the main correlates of pain and functional limitation identified through regression analysis.

### 3.1. Characteristics of the Study Population

The mean age of participants was 45.7 ± 13.5 years (median 48.0; Q1–Q3: 34.0–58.0). Nearly half of the nurses (46.4%) were aged 40–59 years, followed by those aged 21–39 years (32.9%) and ≥60 years (20.7%). Women constituted the overwhelming majority (96.4%). Detailed demographic and occupational characteristics are presented in Table 1.

The mean duration of professional experience was 21.6 ± 13.9 years (median 23.0; Q1–Q3: 9.0–34.0). Short work experience (<10 years) was reported by 27.4% of nurses, medium experience (10–29 years) by 30.7%, and long experience (≥30 years) by 32.8%. Several older nurses had relatively short careers, suggesting later entry into the profession. A strong positive correlation was observed between age and years of professional experience (r = 0.895; *p* < 0.0001), as illustrated in Figure 1.

### 3.2. Health Behavior Inventory Scores

The mean overall HBI score was 82.3 ± 14.4 points (median 83.0; Q1–Q3: 72.5–92.0). Among the four subscales, the lowest mean score was found for Health Practices (19.1 ± 4.4; median 19.0; Q1–Q3: 16.0–22.0), significantly lower than in the remaining domains (*p* < 0.001). Detailed results across all HBI domains are presented in Table 2.

The overall distribution of scores suggests that while nurses demonstrated relatively strong preventive behaviors and positive mental attitudes, their engagement in regular health practices such as exercise, rest, and stress control was noticeably weaker. This pattern identifies an area that may warrant further investigation in occupational health research. Given the central role of health behaviors in occupational well-being, these findings provide important context for understanding the subsequent analyses linking HBI domains with pain indicators.

### 3.3. Laitinen Pain Scale Scores

The average score for Pain Intensity (Indicator 1) was 1.4 ± 0.9 (median 1.0; Q1–Q3: 1.0–2.0); 17.6% of participants reported no pain (score 0), while 0.7% reported unbearable pain (score 4). For Pain Frequency (Indicator 2), the mean was 1.3 ± 0.9 (median 1.0; Q1–Q3: 1.0–2.0); 16.6% reported no pain, and 1.9% reported continuous pain. Analgesic Use (Indicator 3) averaged 1.1 ± 0.7 (median 1.0; Q1–Q3: 1.0–1.0), significantly lower than Indicators 1 and 2 (*p* < 0.001). Motor Activity Limitation (Indicator 4) averaged 1.0 ± 0.9 (median 1.0; Q1–Q3: 0.0–1.0), with 33.4% reporting no limitation and only 0.3% reporting complete dependence.

### 3.4. Correlates of Pain and Related Outcomes

The multivariable analyses showed that professional experience and selected health-behavior domains were associated with several pain-related outcomes. Nurses with 10–29 years of experience were significantly more likely to report pain, frequent pain episodes, and analgesic use than those with less than 10 years of experience (OR 2.94 [1.29–6.64], *p* < 0.01; OR 3.74 [1.54–9.08], *p* < 0.01; OR 2.38 [1.09–5.16], *p* < 0.05, respectively).

Low scores in the Health Practices domain of the HBI were associated with higher odds of pain intensity (OR 2.00 [1.17–3.41], *p* < 0.05), pain frequency (OR 2.20 [1.21–3.99], *p* = 0.01), and analgesic use OR 1.78 [1.09–2.91], *p* < 0.05). For Motor Activity Limitation (Indicator 4), longer work experience (10–29 years: OR 2.78 [1.44–5.37], *p* < 0.01; ≥30 years: OR 3.91 [1.71–8.95], *p* = 0.001) and lower scores in the Positive Mental Attitude domain were significant correlates (OR 1.78 [1.10–2.87], *p* < 0.05).

### 3.5. Logistic Regression Analysis

The analysis included both univariable and multivariable logistic regression models to examine associations with pain and functional limitation. Each model estimated ORs and 95% CIs for the associations between the included variables and positive responses across the four Laitinen Pain Scale indicators—pain intensity, pain frequency, analgesic use, and activity limitation. Variables with *p* < 0.10 in the univariable analyses were considered for inclusion in the corresponding multivariable models, as described in Section 2.6. Odds ratios greater than 1 indicate higher odds of reporting pain or functional limitation relative to the reference category. For the HBI domains, odds ratios greater than 1 for the lower-score category indicate higher odds of the presence of the corresponding LPS outcome (score ≥ 1) compared with the higher-score reference category; they do not indicate a change in the severity of the outcome. Results are summarized in Table 3, Table 4, Table 5 and Table 6.

Across the four regression models, professional experience and selected health-behavior domains showed the most consistent adjusted associations with the LPS outcomes. Nurses with 10–29 years of professional experience had higher odds of pain intensity, pain frequency, and analgesic use, whereas lower scores in the Health Practices domain were associated with higher odds of these same outcomes. Given the small number of male participants, the study was not adequately powered to draw meaningful conclusions regarding sex-related differences.

### 3.6. Correlation Analysis

As shown in Figure 1, age was strongly correlated with years of professional experience (r = 0.895; *p* < 0.0001). The distribution also indicated that some older nurses had relatively short professional experience, suggesting mid-career entry into the nursing profession. This strong correlation confirmed the expected collinearity between age and work experience and justified the exclusion of age from multivariable regression models to avoid bias related to overlapping constructs.

## 4. Discussion

Our study found a high frequency of pain among practicing nurses; more than eight in ten respondents reported pain of varying intensity. These findings are consistent with a European meta-analysis published this year that reported a prevalence of musculoskeletal pain of 87.8% among nurses [4], although it should be noted that our respondents were asked to report any type of pain, not specifically musculoskeletal pain. Work-related exposures typical of nursing have been associated with musculoskeletal pain in previous studies [25]; however, the present study did not assess pain location or etiology. The high frequency of pain observed in this sample underscores the importance of further investigating potentially modifiable occupational and behavioral factors associated with pain among nurses.

In this context, ergonomic, behavioral, and psychosocial interventions may warrant consideration as potential components of occupational health programs for nurses. However, the present cross-sectional study did not evaluate any intervention and cannot establish whether modifying these factors would reduce pain. Longitudinal and interventional studies are needed to determine whether such approaches can reduce pain or improve functional outcomes in this occupational group.

In our sample, more than 80% of nurses reported using analgesics. Prior research indicates that self-medication with painkillers is a common strategy among nurses, often undertaken without visiting a physician [26]. Furthermore, two-thirds of our respondents reported pain-related limitations in daily functioning. Italian data similarly show a comparable proportion of nurses reporting reduced work performance due to pain [9]. Although the present study did not assess pain location or etiology, previous research has identified low-back pain as an important cause of functional limitation among nurses [27]. Presenteeism, or working despite pain, has also been associated with overexertion and symptom exacerbation over time [28].

In our analyses, professional experience was associated with several pain-related outcomes. For pain intensity, pain frequency, and analgesic use, higher odds were observed among nurses with 10–29 years of professional experience compared with those with less than 10 years of experience, whereas this pattern was not consistently observed among those with ≥30 years of experience. One possible hypothesis for this pattern is the healthy worker effect, whereby nurses with more severe or persistent health problems may be less likely to remain in active employment. However, this mechanism was not assessed in the present study and should therefore not be considered an explanation of the observed association. Previous studies have reported that musculoskeletal pain contributes to disability and sickness absence among nurses [3,29]. Sickness absence may also reduce staffing availability and increase workload among remaining staff [30], while workforce attrition has been associated with factors such as burnout and staffing shortages [31]. These observations provide broader occupational context but do not establish that a healthy worker effect occurred in our sample. Longitudinal studies are needed to determine whether selective retention in the workforce contributes to the observed relationship between professional experience and pain.

A second key correlate was lower scoring in the Health Behavior Inventory domain of Health Practices. Previous studies have highlighted suboptimal health behaviors among employed nurses [32]. In the present study, nurses scored lowest in this domain, significantly lower than in other HBI areas, indicating specific deficits in routine preventive checks, physical activity, and stress management. In work by Keely R., nurses scored lower than community referents precisely in physical activity and stress-coping behaviors, a finding at odds with public expectations that nurse’s model healthy lifestyles [33].

The literature simultaneously points to the frequent non-engagement in physical activity among nurses [34,35] and to the association between pain and lack of habitual physical activity [17]. Other studies link low-back pain in nurses with job-related stress [18]. Collectively, these findings suggest that health-related behaviors warrant further investigation as potential correlates of pain among working nurses. Given the cross-sectional design, it remains unclear whether less favorable health practices contribute to pain, whether pain adversely affects these behaviors, or whether both are influenced by other factors.

For the Laitinen indicator capturing mobility limitation (Indicator 4), lower scores in positive mental attitude (HBI Area 3) emerged as an additional correlate beyond years of experience. Disability and pain are tightly intertwined with negative affect, which can consolidate and deepen physical limitations through reciprocal reinforcement [19]. Causality cannot be established from our cross-sectional data. It is plausible that, in some nurses, poorer mental well-being, e.g., fear of pain, promotes movement avoidance and an energy-conserving functional style [36], while conversely, everyday functional constraints may drive fatigue and pain, which then negatively affect mood [37]. In a study among nurses in China, individuals with chronic pain more frequently reported deterioration in mental health [27].

Sex was not statistically associated with pain outcomes in our analyses; however, this finding should be interpreted with considerable caution. Women constituted 96.4% of the sample, whereas only 3.6% (n = 21) of participants were men. Consequently, the study had very limited ability to assess sex-related differences, and the absence of statistically significant associations should not be interpreted as evidence that such differences do not exist. Previous studies have reported higher pain prevalence among female nurses [38], but reliable sex-specific comparisons could not be made in the present sample. Workplace category was not significantly associated with pain outcomes in the multivariable analyses.

The findings may help identify areas for further occupational health research, particularly health practices, work-related factors, and psychological well-being. Ergonomic measures, safe patient-handling practices, physical activity, stress management, and mental health support have been discussed in the broader literature as potentially relevant components of occupational health programs for nurses [39,40]. However, their effectiveness in reducing pain cannot be inferred from the present study and should be evaluated in appropriately designed longitudinal and interventional studies.

Future studies should use longitudinal designs to clarify the temporal relationships between health behaviors, professional experience, and pain. Recruitment across multiple regions and the use of more representative sampling strategies would improve the generalizability of the findings, while adequate representation of both sexes would allow more reliable assessment of sex-related differences. Future research should also characterize pain location, duration, and etiology and, where analytically appropriate, retain the information provided by the original scale categories. Finally, intervention studies are needed to determine whether modification of occupational or health-behavior factors can reduce pain or improve functional outcomes among nurses.

## 5. Limitations

This study has several limitations that should be considered when interpreting the findings. First, the cross-sectional design precludes conclusions regarding temporality or causality. The observed relationships between professional experience, health behaviors, and pain represent associations only, and the direction of these relationships cannot be established. In particular, less favorable health practices may contribute to pain, pain may adversely affect health behaviors, or both may reflect other underlying factors. Longitudinal and interventional studies are needed to clarify these relationships.

Second, the study relied on self-reported measures of pain, analgesic use, activity limitation, and health behaviors, which may be subject to recall and reporting bias. In addition, the Laitinen Pain Scale assessed pain broadly without establishing its anatomical location, etiology, or duration. The findings should therefore not be interpreted specifically as reflecting musculoskeletal, work-related, or chronic pain, and comparisons with studies focusing on these specific pain conditions should be made with caution.

Third, participants were recruited using non-probability convenience sampling in southeastern Poland, and a response rate could not be calculated because the participating institutions did not systematically record the number of questionnaires distributed or potentially eligible nurses invited to participate. Selection and non-response bias therefore cannot be excluded, and the findings should not be considered nationally representative of nurses in Poland. The possibility of a healthy worker effect should also be considered, as nurses with more severe health problems may be less likely to remain in active employment; however, this mechanism was not directly assessed in the present study.

Fourth, the marked sex imbalance limited the assessment of sex-related differences. Only 21 participants (3.6%) were male, resulting in limited statistical power for sex-specific comparisons. Although the predominance of women in the sample broadly reflects the strongly female-dominated nursing workforce in Poland, the very small number of male participants precluded reliable assessment of sex-related differences. Consequently, the absence of statistically significant associations with sex should not be interpreted as evidence that sex-related differences do not exist.

Fifth, several analytical decisions should be considered when interpreting the regression findings. HBI domain scores were dichotomized at the sample median, and the four LPS outcomes were analyzed as score 0 versus score ≥1. Although these operationalizations provided consistent comparisons, dichotomization resulted in loss of information, and the HBI median categories represent relative groups within this sample rather than validated clinical thresholds. Similarly, the regression models address the presence versus absence of each LPS outcome rather than differences in its severity. Candidate variables were also selected using a univariable screening threshold of *p* < 0.10. This was used as an exploratory screening criterion rather than as evidence of statistical significance, but such data-driven selection may contribute to model instability and incomplete control of confounding.

Sixth, age and professional experience were strongly correlated and were therefore not included simultaneously in the multivariable models; professional experience was retained as the more directly interpretable occupational characteristic. Consequently, residual confounding by age cannot be excluded, and associations with professional experience should not be interpreted as effects independent of age. Information on chronic medical conditions and other relevant clinical factors that could potentially influence both pain and health-related behaviors was not collected. Therefore, residual confounding by underlying health status cannot be excluded. In addition, no separate a priori sample-size calculation was performed for each logistic regression model, and additional formal goodness-of-fit diagnostics were not reported. These considerations warrant appropriate caution regarding the stability and interpretation of the adjusted estimates.

Despite these limitations, the study included a relatively large sample of 580 practicing nurses and assessed multiple dimensions of pain and health-related behaviors using standardized instruments. The analysis considered a range of sociodemographic, occupational, and behavioral characteristics and quantitatively assessed multicollinearity among variables retained in the regression models. By examining individual HBI domains alongside separate dimensions of the LPS, the study provides a differentiated assessment of behavioral and occupational characteristics associated with pain and identifies questions that can be addressed in future longitudinal and intervention studies.

## 6. Conclusions

This study found a high frequency of pain among practicing nurses in southeastern Poland and identified cross-sectional associations between selected pain-related outcomes, professional experience, and specific health-behavior domains. Nurses with 10–29 years of professional experience had higher odds of pain intensity, pain frequency, and analgesic use, while lower Health Practices scores were associated with higher odds of these same outcomes. Lower Positive Mental Attitude scores were associated with activity limitation. These findings should be interpreted as associations rather than causal relationships. They identify areas for further occupational health research and support the need for longitudinal and interventional studies to determine whether modification of occupational or health-behavior factors can reduce pain or improve functional outcomes among nurses.

## Figures and Tables

**Figure 1:**
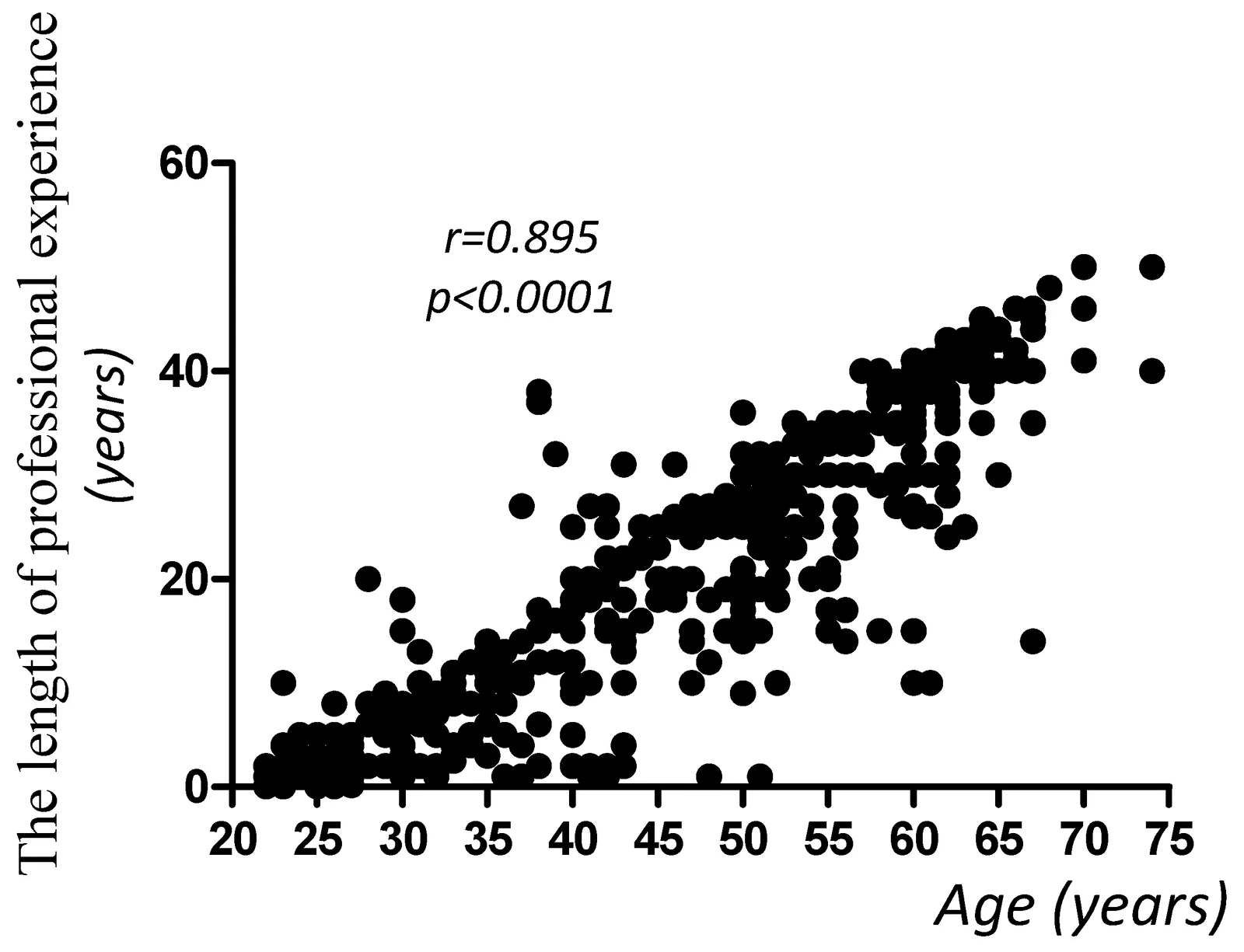
Correlation between age and years of professional experience among nurses (n = 580).

**Table 1:** Sociodemographic and occupational characteristics of participating nurses (n = 580).

Variable	Category	n (%)
Age group (years)	21–39	191 (32.9)
	40–59	269 (46.4)
	≥60	120 (20.7)
Sex	Female	559 (96.4)
	Male	21 (3.6)
Education	Medical college	122 (21.0)
	Bachelor’s degree	261 (45.0)
	Master’s degree	197 (34.0)
Workplace	Hospital	393 (67.8)
	Outpatient clinic	108 (18.6)
	Long-term care facility	46 (7.9)
	Other (sanatoria, nurseries)	13 (2.2)
	Not specified	20 (3.5)
Years of professional experience	<10	159 (27.4)
	10–29	178 (30.7)
	≥30	190 (32.8)
Additional employment	Yes	261 (45.0)
	No	319 (55.0)

Note: Data are presented as n (%).

**Table 2:** Health Behavior Inventory scores among participating nurses (n = 580).

HBI Subscale	Mean ± SD	Median (Q1–Q3)
Proper eating habits	20.7 ± 4.4	21.0 (18.0–24.0)
Preventive behaviors	21.4 ± 4.4	22.0 (19.0–25.0)
Positive mental attitude	21.1 ± 4.2	22.0 (18.0–24.0)
Health Practices	19.1 ± 4.4	19.0 (16.0–22.0)

**Table 3:** Correlates of positive responses (pain presence) on the Laitinen Pain Scale—Indicator 1: Pain Intensity (n = 580).

Parameter	Positive Responses n (%)	Univariable Analysis *p*	Multivariable OR (95% CI), *p*
Sex	Females 447 (81.9%)/Males 17 (85.0%)	0.714	—
Age group	21–39 (72.5%), 40–59 (87.3%), ≥60 (85.6%)	<0.001	I vs. II OR 1.25 (0.56–2.80), *p* = 0.595
Education	Medical college 88.2%/Bachelor 79.6%/Master 81.3%	0.123	—
Place of work	Hospital 79.4%/Outpatient 87.7%/LTC 91.3%	<0.05	I vs. II OR 1.86 (0.90–3.83), *p* = 0.092
Additional work (yes vs. no)	87.1% vs. 77.7%	<0.01	OR 0.69 (0.40–1.20), *p* = 0.185
Years of work (<10 vs. 10–29/≥30)	68.4% vs. 88.5%/85.1%	<0.001	II vs. I OR 2.94 (1.29–6.64), *p* < 0.01
HBI Area 4—Health Practices	≤median 85.5% vs. >median 77.9%	<0.05	OR 2.00 (1.17–3.41), *p* < 0.05

Note: OR—odds ratio; CI—confidence interval; LTC—long-term care; HBI—Health Behavior Inventory. Multivariable models included variables selected using the univariable screening procedure described in the Statistical Analysis Section.

**Table 4:** Correlates of positive responses on the Laitinen Pain Scale—Indicator 2: Pain Frequency (n = 580).

Parameter	Positive Responses n (%)	Univariable *p*	Multivariable OR (95% CI), *p*
Sex (F/M)	453 (83.0%)/17 (85.0%)	0.812	—
Age (21–39/40–59/≥ 60)	75.1/88.0/84.8	<0.05	I vs. II OR 1.15 (0.48–2.73), *p* = 0.761
Education (Medical college/Bachelor/Master)	88.2/81.2/82.3	0.225	—
Place of work (Hospital/Outpatient/LTC)	80.9/87.7/91.3	0.078	I vs. II OR 1.68 (0.80–3.50), *p* = 0.168
Additional work (yes/no)	86.2/80.4	0.089	OR 0.95 (0.54–1.65), *p* = 0.847
Years of work (<10/10–29/≥ 30)	71.3/90.6/84.0	<0.0001	II vs. I OR 3.74 (1.54–9.08), *p* < 0.01
HBI Area 3—Positive mental attitude	≤median 85.7/>median 79.3	<0.05	OR 1.24 (0.67–2.29), *p* = 0.494
HBI Area 4—Health practices	≤median 87.2/>median 78.2	<0.01	OR 2.20 (1.21–3.99), *p* = 0.010

Note: OR—odds ratio; CI—confidence interval; LTC—long-term care; HBI—Health Behavior Inventory. Multivariable models included variables selected using the univariable screening procedure described in the Statistical Analysis Section.

**Table 5:** Correlates of positive responses on the Laitinen Pain Scale—Indicator 3: Analgesic Use (n = 580).

Parameter	Positive Responses n (%)	Univariable *p*	Multivariable OR (95% CI), *p*
Sex (F/M)	445 (81.5%)/17 (85.0%)	0.692	—
Age (21–39/40–59/≥60)	73.0/86.9/83.9	<0.001	II vs. I OR 1.50 (0.69–3.25), *p* = 0.309; III vs. I OR 1.41 (0.51–3.89), *p* = 0.507
Education (Medical college/Bachelor/Master)	85.7/80.0/81.3	0.408	—
Place of work (Hospital/Outpatient/LTC)	81.5/81.1/91.3	0.242	—
Additional work (yes/no)	84.3/79.4	0.163	—
Years of work (<10/10–29/≥30)	70.6/88.0/83.5	<0.001	II vs. I OR 2.38 (1.09–5.16), *p* < 0.05; III vs. I OR 1.72 (0.67–4.42), *p* = 0.260
HBI Area 2—Preventive behaviors	≤median 84.6/>median 77.8	<0.05	OR 1.35 (0.83–2.20), *p* = 0.224
HBI Area 4—Health practices	≤median 85.5/>median 77.1	<0.01	OR 1.78 (1.09–2.91), *p* < 0.05

Note: OR—odds ratio; CI—confidence interval; LTC—long-term care; HBI—Health Behavior Inventory. Multivariable models included variables selected using the univariable screening procedure described in the Statistical Analysis Section.

**Table 6:** Correlates of positive responses on the Laitinen Pain Scale—Indicator 4: Motor Activity Limitation (n = 580).

Parameter	Positive Responses n (%)	Univariable *p*	Multivariable OR (95% CI), *p*
Sex (F/M)	361 (66.1%)/11 (55.0%)	0.304	—
Age (21–39/40–59/≥ 60)	51.9/70.7/77.1	<0.001	II vs. I OR 0.95 (0.50–1.82), *p* = 0.878
Education (Medical college/Bachelor/Master)	74.8/62.4/64.6	0.057	I vs. II OR 0.77 (0.41–1.44), *p* = 0.419
Place of work (Hospital/Outpatient/LTC)	64.8/70.8/73.9	0.284	—
Additional work (yes/no)	70.5/59.8	<0.05	OR 0.85 (0.55–1.30), *p* = 0.450
Years of work (<10/10–29/≥30)	44.9/69.8/77.1	<0.001	II vs. I OR 2.78 (1.44–5.37), *p* < 0.01
HBI Area 3—Positive mental attitude	≤median 71.1/>median 58.2	<0.01	OR 1.78 (1.10–2.87), *p* < 0.05
HBI Area 4—Health practices	≤median 68.1/>median 63.0	<0.001	OR 1.10 (0.68–1.75), *p* = 0.719

Note: OR—odds ratio; CI—confidence interval; LTC—long-term care; HBI—Health Behavior Inventory. Multivariable models included variables selected using the univariable screening procedure described in the Statistical Analysis Section.

## Data Availability

The data that support the findings of this study are avail-able from the corresponding authors upon reasonable request due to privacy.
